# Supplementary material for: Predictors of Preeclampsia in the First Trimester in Normotensive and Chronic Hypertensive Pregnant Women
Source: J Clin Med. 2023 Jan 11;12(2):579. doi: 10.3390/jcm12020579 (PMC9865932; doi:10.3390/jcm12020579)
Supplement: Supplementary file 1 [file jcm-12-00579-s001.zip › jcm-2081927-supplementary.pdf]

Supplementary material:

Tables

Table S1: Office pulse pressure values in the first trimester in normotensive and chronic hypertensive pregnant women.

	Normotensive			cHT		
	Unaffected (N=116)	PE (N=8)	*p**	Unaffected (N=9)	sPE (N=11)	*p*
Office PP (mmHg)	42.7 ± 7.4	44.7 ± 4.9	0.467	48.7 ± 10.2	44.4 ± 7.2	0.279

PP: Pulse pressure, cHT: chronic hypertensive, PE: preeclampsia, sPE: superimposed preeclampsia. * After adjusting for BMI, history of PE in previous pregnancies and Caucasian race.

Table S2. Ambulatory pulse pressure values in the first trimester in normotensive and chronic hypertensive pregnant women.

	Normotensive			cHT		
	Unaffected (N=116)	PE (N=8)	*p**	Unaffected (N=9)	sPE (N=11)	*p*
Daytime PP (mmHg)	42.4 ± 5.7	44.8 ± 4.2	0.231	51.1 ± 8.1	46.9 ± 8.6	0.280
Nighttime PP (mmHg)	41.9 ± 6.3	44.5 ± 5.1	0.268	48.1 ± 9.6	48.1 ± 11.7	0.989
24-hour PP (mmHg)	42.2 ± 5.6	44.7 ± 4.4	0.222	50.0 ± 8.6	47.2 ± 9.2	0.506

PP:Pulse pressure, cHT: chronic hypertensive, PE: preeclampsia, sPE: superimposed preeclampsia. * After adjusting for BMI, history of PE in previous pregnancies and Caucasian race.

Figures:

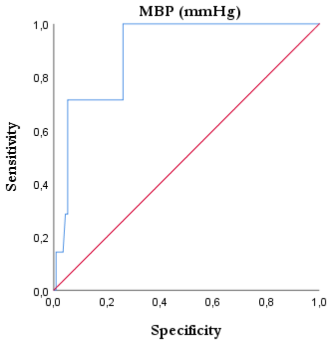

Figure S1. ROC curve for the prediction of PE in normotensive pregnant women according to office MBP.

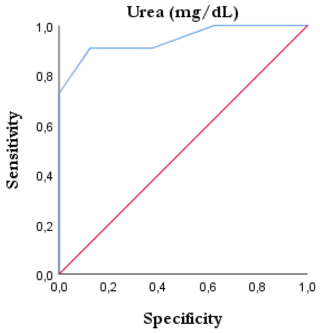

Figure S2. ROC curve for the prediction of sPE HTN in chronic hypertensive pregnant women according to plasma urea.
